# Identification, screening, and comprehensive evaluation of novel thrombin inhibitory peptides from the hirudo produced using pepsin

**DOI:** 10.3389/fphar.2024.1460053

**Published:** 2024-11-21

**Authors:** Xiaoyu Chai, Fulu Pan, Qianqian Wang, Xinyu Wang, Xueyan Li, Dongying Qi, Zirong Yi, Huan Liu, Jing Zhang, Yiming Zhang, Yanli Pan, Yang Liu, Guopeng Wang

**Affiliations:** ^1^ Department of Chemistry of Traditional Chinese Medicine, School of Chinese Materia Medica, Beijing University of Chinese Medicine, Beijing, China; ^2^ Institute of Information on Traditional Chinese Medicine, China Academy of Chinese Medical Sciences, Beijing, China; ^3^ Zhongcai Health (Beijing) Biological Technology Development Co., Ltd., Beijing, China

**Keywords:** hirudo, thrombin, *in silico* analysis, molecular dynamics simulations, peptides

## Abstract

**Purpose:**

The inhibition of thrombin has proven to be an efficacious therapeutic approach for managing cardiovascular disease (CVD), with widespread implementation in clinical settings. Oral ingestion of peptides and protein drugs is influenced by gastrointestinal digestive enzymes. We aimed to evaluate the thrombin inhibitory properties of hirudo hydrolysates (HHS) produced by pepsin and propose a comprehensive approach to screen and evaluate thrombin inhibitors.

**Methods:**

We evaluated the *in vitro* inhibitory properties of the hirudo extract, both before and after hydrolysis with pepsin, toward thrombin. We screened for the most potent thrombin inhibitory peptide (TIP) using nano liquid chromatography-tandem mass spectrometry (Nano LC-MS/MS) coupled with *in silico* analysis. Next, we employed the thrombin inhibition activity IC_50_ to investigate the interaction between TIP and thrombin, and conducted *in vitro* evaluations of its anticoagulant effects (APTT, TT, PT), as well as its ability to inhibit platelet aggregation. Furthermore, we utilized UV-Vis spectroscopy to explore structural changes in thrombin upon binding with TIP and employed molecular dynamics simulations to delve deeper into the potential atomic-level interaction modes between thrombin and TIP.

**Results:**

The retention rate of thrombin inhibition for HHS was found to be between 60% and 75%. A total of 90 peptides from the HHS were identified using LC-MS/MS combined with *de novo* sequencing. Asn-Asp-Leu-Trp-Asp-Gln-Gly-Leu-Val-Ser-Gln-Asp-Leu (NDLWDQGLVSQDL, P1) was identified as the most potent thrombin inhibitory peptide after *in silico* screening (molecular docking and ADMET). Then, the *in vitro* study revealed that P1 had a high inhibitory effect on thrombin (IC_50_: 2,425.5 ± 109.7 μM). P1 exhibited a dose-dependent prolongation of the thrombin time (TT) and a reduction in platelet aggregation rate. Both UV-Vis spectroscopy and molecular dynamics simulations demonstrated that P1 binds effectively to thrombin.

**Conclusion:**

Overall, the results suggested that HHS provides new insights for searching and evaluating potential antithrombotic compounds. The obtained P1 can be structurally optimized for in-depth evaluation in animal and cellular experiments.

## Graphical Abstract

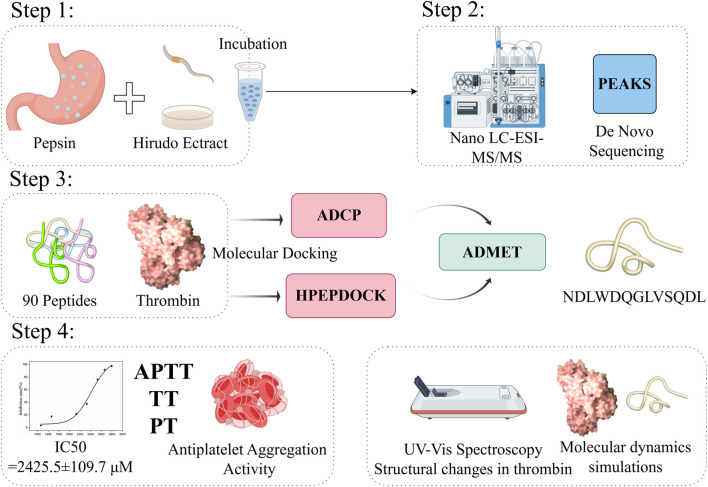

## 1 Introduction

For cardiovascular disease (CVD) is the primary cause of global mortality and significantly impacts quality of life (Mensah et al., 2019). Thrombosis, the formation of blood clots within blood vessels, is the underlying cause of CVD (Cicha, 2015). The process of blood clot formation is intricately linked to the activation of coagulation cascade reactions, platelet aggregation, and fibrinogen formation (Garmo et al., 2024). Various treatment options for thrombosis include anticoagulation, antiplatelet aggregation, and antifibrosis therapies. Thrombin, a critical enzyme in the blood coagulation cascade, plays a central role in both hemostasis and thrombosis. Dysregulation of thrombin activity has been implicated in a variety of pathological conditions, particularly thromboembolic disorders, highlighting its potential as a target for therapeutic intervention. While synthetic drugs like aspirin, warfarin, dabigatran, and rivaroxaban are commonly used to treat thrombotic disorders, clinical studies have demonstrated that these chemically synthesized medications often lead to adverse side effects (Li et al., 2020). For example, aspirin, a widely used antiplatelet drug, has been associated with gastrointestinal bleeding, nephritis, and other negative effects with prolonged use (Bhatia et al., 2023); Warfarin reduces active procoagulant clotting coagulation factors II, VII, IX and X by interfering with vitamin K but it is higher rates of gastrointestinal bleeding and continuous monitoring is needed (Ingason et al., 2023). Furthermore, oral anticoagulant such as Dabigatran and Rivaroxaban carries the potential risk of severe or life-threatening hemorrhagic complications.

Therefore, it is important to find safe and effective anticoagulants for the treatment of thrombotic disorders. Natural products have been acknowledged for their significant role as a source of pharmacologically active compounds (Atanasov et al., 2021). Natural traditional Chinese medicine leeches can promote blood circulation and remove blood stasis (Qiu et al., 2019). Recent medical studies have demonstrated their diverse effects, including anticoagulation, antiplatelet aggregation, and anti-inflammatory properties, making them a common treatment for cardiovascular diseases and other ailments (Qiu et al., 2019; Han et al., 2020; Montinari and Minelli, 2022). It is worth noting that there are over 680 species of leeches worldwide (Qiu et al., 2019). However, only three species, namely, Whitmania pigra Whitman, Hirudo nipponica Whitman and Whitmania acranulata Whitman, are recognized in the current Chinese Pharmacopoeia as the medicinal sources of leeches (Li et al., 2024a). Among the components of leeches, leech peptides are the most studied active compounds (Liao et al., 2023). Hirudin, a naturally occurring peptide extracted from the saliva of hematophagous leeches, is recognized as the most potent natural thrombin inhibitor to date. However, hirudin is easily degraded when orally administered by pepsin (Dong et al., 2016). In recent years, new peptides have been reported to potentially possess anticoagulant activity, such as SYELPDGQVITIGNER (Huang et al., 2020) and WP-77 (Zhang et al., 2022). Naoxuekang Drop Pill, composed of traditional Chinese medicine hirudo extract, has shown inhibitory effects on thrombin, although the specific anticoagulant components remain to be fully elucidated (Chai et al., 2024).

The development of oral formulations for peptide- or protein-based drugs poses challenges due to proteolytic degradation in the gastrointestinal tract (Davies et al., 2017). Interestingly, we found that hirudo extracts exhibited sustained activity following incubation with pepsin, while a significant decrease in activity was noted after incubation with trypsin. This suggests that the hirudo extract may contain components resistant to degradation by pepsin, or that its degraded components retain activity. Consequently, hirudo peptides hydrolyzed by pepsin show promise as potential lead compounds for screening anticoagulant properties.

Based on the above analysis, we aimed to explore the potential thrombin inhibitory activity of hirudo extract hydrolysate prepared using pepsin, aiming to release thrombin-inhibiting peptides that have a higher potential for oral absorption. We screened the most potent thrombin inhibitory peptide from peptide mixtures using liquid chromatography-tandem mass spectrometry (LC-MS/MS) coupled with *in silico* analysis. Subsequently, we proposed an integrated strategy to evaluate the potential inhibitory activity against thrombin, including activity analysis (IC_50_, Anticoagulant activity, and platelet aggregation activity) and structural analysis (UV-Vis and molecular dynamics simulation).

## 2 Materials and methods

### 2.1 Chemicals and reagents

Recombinant hirudin was purchased from Tianjin Xiensi Biological Technology Co., Ltd. (Tianjin, China); The extract of hirudo was provided by Beijing Kang’er Fu pharmaceutical Co., Ltd.; Pepsin, thrombin, chromogenic substrate S-2238 and adenosine diphosphate (ADP) were purchased from Shanghai Yuanye Bio-Technology Co., Ltd. (Shanghai, China); Prothrombin Time (PT) reagent, Activated partial thromboplastin time (APTT) reagent, calcium chloride, and Thrombin Time (TT) reagent were purchased from Stago Co., Ltd.; The synthetic peptides were obtained from Shanghai Top-Peptide Biotechnology Co., Ltd (Shanghai, China).

Sprague–Dawley rats (males, 200–250 g) were procured from Spfanimals Laboratory Animal Technology Co., Ltd. (Beijing, China), with a license number of SCXK (Jing)2024-0001.

### 2.2 Extraction method and pepsin treatment

150 g of hirudo were finely chopped and soaked in water for a duration of 4 h. Subsequently, they underwent water reflux extraction three times: initially for a period of 2 h, followed by two additional extractions lasting 1.5 h each. The resulting extracts were combined, filtered, and concentrated to a relative density falling within the range of 1.20–1.25 (at a temperature of 50°C). Following a cooling period, ethanol was introduced to achieve a 70% alcohol content within the mixture. The solution was thoroughly agitated, allowed to settle for a duration of 24 h, and subsequently filtered. The ethanol was then evaporated until no residual alcohol taste remained, after which the mixture was left to stand for an additional 24 h before undergoing a second filtration process. The resulting filtrate was further concentrated and dried.

Simulated gastric fluid: 16.4 mL of dilute hydrochloric acid was added about 800 mL of water and 10 g of pepsin, and the mixture was diluted with water to 1,000 mL. Then, the hirudo extracts were suspended in a simulated gastric fluid (17.5 mg/mL), and the digestion process was carried out in a water bath at 37°C. After incubation for 5, 15, 30, 60 min, the reaction was terminated by heating it in a water bath at 100°C for 10 min and adjusting the pH value to 7.0 with 1.0 M NaOH solution. Using hirudin as a control, the thrombin inhibitory activity was measured before and after incubation with pepsin.

### 2.3 *In Vitro* thrombin inhibition activity

Thrombin activity was determined in accordance with a reported method (Wang et al., 2020; Zhen et al., 2024) with several modifications due to the influence of the drug’s inherent color. To a 1.5 mL centrifuge tube, add 200 µL of Tris-HCl buffer (pH 7.4, 0.308 mmol L^-1^ NaCl), 80 µL of hirudo herbal preparation sample solution, and 20 µL of thrombin (final concentration 2 nmol L^-1^). Following thorough mixing, introduce 20 µL of thrombin chromogenic substrate S-2238 (final concentration 60 μmol L^-1^), to initiate the thrombin-catalyzed hydrolysis reaction of S-2238. Use 80 µL of Tris-HCl buffer in place of the sample solution for the blank control. Incubate at 37°C for 10 min, then add 80 μL of 1% sodium dodecyl sulfate (SDS) solution to halt the reaction. After centrifugation at 10,000 rpm for 10 min, take the supernatant to measure the peak area of pNA, and calculate the thrombin inhibition rate using formula:

$$Thrombin\;inhibition=1\;–\left(\;A\;sample\;/\;A\;control\right)\times 100$$

Where A sample and A control are the peak areas of pNA with and without the addition of hirudo herbal preparation in the thrombin inhibition reaction, respectively. When determining the IC_50_ (the concentration of inhibitor required to inhibit 50% of thrombin activity) of P1 against thrombin, the concentrations of P1 were set at a series of concentrations. The other reaction conditions were the same as those described above.

Sample analysis was carried out using Shimadzu HPLC with UV detector. Chromatographic conditions: Column: Xbridge C_18_ column (Waters, 4.6 mm × 250 mm, 5 μm); Mobile phase: Acetonitrile-water (30:70 v:v); Flow rate: 1.0 mL/min; Column temperature: 35°C; Detection wavelength: 365 nm; Injection volume: 10 μL. The established analytical method has undergone method validation, proving it can accurately determine the test samples.

### 2.4 Identification of peptide sequences by nano LC-ESI-MS/MS

Take an appropriate amount of samples, add 1 mM DTT solution to the final concentration of 10 mmol/L, and incubate at 56°C for 1 h. Then, iodoacetamide was added to achieve a final concentration of 55 mmol/L, followed by incubation for 40 min at room temperature in the dark. After digestion, peptides were desalted using a self-priming desalting column, and the solvent was evaporated in a vacuum centrifuge at 45°C. The dried samples were dissolved in 0.1% formic acid (FA) and used for LC-MS analysis.

Hirudo hydrolysate produced by pepsin was submitted to nano-liquid chromatography using EASY-nLC 1,200 (Thermo Scientific, San Jose, CA, United States) coupled with Q Exactive™ Hybrid Quadrupole-Orbitrap™ Mass Spectrometer (Thermo Scientific,San Jose, CA, United States). The analysis conditions were as follows: analytical column—Acclaim PepMap RPLC C18 150 μm × 150 mm (1.9 µm, 100 Å; Thermo Scientific,San Jose, CA, United States); mobile phase A was 0.1% FA (v/v) and mobile phase B was 0.1% FA in 80% ACN (v/v); gradient—0–2 min at 4%–8% B, 2–45 min at 8%–28% B, 45–55 min at 28%–40% B, 55–56 min at 40%–95% B and 56–66 min at 95% B; and flow rate—600 nL/min. Full scan MS was performed using an Orbitrap for first stage scanning in the range of m/z 100–1,500 with resolution set to 70,000. The maximum ion introduction time was 100 ms and the automatic gaincontrol (Automatic gain control, AGC) was set to 3 × 10^6^. The scanning resolution was set to 17,500, the maximum ion introduction time for MS/MS was 50 ms, the AGC control was set at 1.0 × 10^5^.

Data analysis and *de novo* sequencing were performed using PEAKS studio version 10.6(Bioinformatics Solutions Inc., Waterloo, ON, Canada).

### 2.5 Molecular docking

The three-dimensional structure of thrombin (PDB code: 2BVR) was retrieved from the Protein Data Bank. The protein structure was prepared by removing heteroatoms and water molecules using PyMOL (version 3.0.1).

For HPEPDOCK, the peptides and 2BVR protein were uploaded as ligand and receptor molecules, respectively, to the HPEPDOCK web server. HPEPDOCK was used for the flexible and blind global docking experiments. For each docking, the HPEPDOCK server generated 100 docking poses, which were subsequently ranked based on their binding energies. From the results page, we could review the binding energies and docking poses of the top ten models. The top-ranked docking pose, presenting the most favorable binding energy, was then chosen for further analysis.

For ADCP, the structures of peptides were generated with a Python package named PeptideConstructor (Tien et al., 2013). Hydrogen atoms were subsequently added using the Ambertools Reduce module prior to energy minimization using the Tinker software, employing the CHARMM22 force field to minimize energy with an RMS parameter set to 0.5. For docking to thrombin, a binding site within a 24 × 15 × 32 Å box centered at coordinates x: 12, y: −17, and z: 18 was explored. Docking modes were generated and assessed based on affinity energy values.

For molecular docking data processing, the first step is to standardize the results obtained and establish a common docking assessment system. In our manuscript, the results of the two sets of molecular docking were used for normalization. The top ten compounds in the list will be used for the analysis of ADMET.

### 2.6 ADMET analysis

The screened peptides were translated into simplified molecular input line entry specification (SMILES) by the PepSMI online server (https://www.novoprolabs.com/tools/convert-peptide-to-smiles-string). In accordance with SMILES, admetSAR 2.0 (http://lmmd.ecust.edu.cn/admetsar2/) (Hongbin Yang et al., 2019) was used to predict the ADMET properties of the peptides (Renjuan et al., 2022). A compound with higher absorption potential will be used for subsequent activity verification.

### 2.7 Determination of PT, APTT and TT

The whole blood was collected from SD rats via the abdominal aorta. A disposable blood collection needle with a silicone tube was used as the catheter, and 3.8% sodium citrate anticoagulant (anticoagulant: whole blood = 1:9) was used to prevent coagulation. The collected blood was gently inverted 5-8 times and centrifuged at 4,000 rpm for 10 min to obtain the plasma from the upper layer.

APTT, PT, TT were determined by diagnostic kits (Stago, France), using Compact Max Automated Coagulation Analyzer (Stago, France). To measure the coagulation time at different concentrations of P1 and ensure the same volume of coagulation system, each sample was adjusted to the same final volume using Tris-HCl (pH = 7.4, 0.308 mM NaCl). The samples were then subjected to the coagulation assays as per the manufacturer’s instructions.

### 2.8 The antiplatelet aggregation activity of peptide

The blood was collected as described previously. To obtain platelet-rich plasma (PRP), the blood was centrifuged at room temperature for 10 min at 800 rpm. Subsequently, to prepare platelet-poor plasma (PPP), the PRP was further centrifuged at 3,000 rpm for 10 min at room temperature. PPP and pure water were used as the negative control and blank control, respectively. 10 μL of the sample was mixed with 50 μL of either PRP or the negative control (PPP) in a centrifuge tube. The contents were then vortexed to ensure homogeneity and incubated at 37°C for 3 min. Subsequently, 45 μL of this mixture was transferred into a 96-well plate, followed by the addition of 5 μL of ADP (adenosine diphosphate, 50 μM) as an inducer to initiate platelet aggregation. The aggregation process was monitored turbidimetrically at 405 nm using a MULTISKAN FC microplate reader (Thermo Fisher Scientific). The maximum rate of aggregation within a 10-minute period was determined and recorded for each sample.

### 2.9 UV-vis spectroscopy study

The UV-vis spectra were measured using a TU-1810 spectrophotometer (Puxi, Beijing, China). P1 and thrombin were first dissolved separately in Tris-HCl buffer (pH = 7.4, 0.308 mM NaCl). 1 mL of P1 solution (concentration) and 2 mL of thrombin (5 µM) solution were mixed and incubated at 37°C for 10 min. The mixture was then placed in a UV spectrophotometer to measure the spectra at 190–350 nm. All spectra were collected in a 1 cm light-path quartz cell (Li et al., 2024b).

### 2.10 Molecular dynamic simulation studies

The protein structure of thrombin (PDB code: 2BVR) was obtained from the Protein Data Bank database (https://www.rcsb.org/). The structure of the peptide-protein complex was constructed using AutoDock CrankPep, while MD simulations were carried out using software GROMACS 2020.03 (Abraham et al., 2015; Páll et al., 2015), and water molecules were used TIP3P model (Mark and Nilsson, 2001; Bogunia and Makowski, 2020). The steepest descent method was used to simulate and optimize the system to reduce unreasonable contacts or atomic overlap in the entire system. Subsequently, a restricted MD simulation was performed to balance the system under the canonical and isothermal isobaric ensemble, making the simulation system fully pre-balanced. The Verlet leapfrog algorithm was used to solve Newton’s equation of motion, with the integration step set to 2 fs. In the calculation process, the Lennard-Jones function was plotted to calculate the van der Waals (VDW) force, and the non-bond cutoff distance was set to 1.2 nm; the bond length of all atoms were constrained by the LINCS algorithm, and the particle mesh Ewald (PME) method was used to calculate the short-range electrostatic interactions (Schulz et al., 2009). Periodic boundary conditions were used in the simulation. All MD simulations were performed under isothermal and isobaric ensemble, with a temperature of 310.15 K and a pressure of 1 atm. The temperature and pressure were controlled by the V-rescale and Parrinello-Rahman methods. The temperature and pressure coupling constants were set to 0.1 ps and 0.5 ps, respectively. The molecular dynamics simulation duration was set to 200 ns. Visualization and graphics were generated using PyMOL software (https://pymol.org/2/support.html).

### 2.11 Statistical analysis

All tests were performed in triplicate. Results are presented as means ± standard deviations (SD). In the analysis performed using GraphPad Prism version 10.2.3 (GraphPad Software, Inc., La Jolla, CA, United States), a one-way analysis of variance (ANOVA) was used to compare the differences between the test group and the blank or control group. A *p*-value of less than 0.05 (*), 0.01 (**), or 0.001 (****) was considered statistically significant, indicating a significant difference compared to the blank group.

## 3 Results and discussion

### 3.1 Anticoagulant activity after hydrolysis by pepsin

**FIGURE 1:**
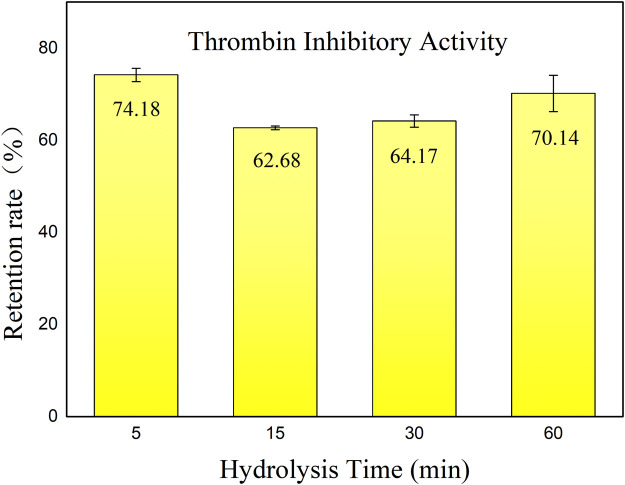
Retention rate of thrombin inhibitory activity of hirudo peptides. Data are represented as means ± SD (n = 3). The *x*-axis represents the various incubation times with pepsin, while the *y*-axis indicates the residual thrombin inhibitory activity of HHS following incubation, as compared to the non-incubated condition.

In this study, anticoagulant activity (thrombin inhibition activity) of both HHS and hirudin before and after incubation with pepsin was determined. Figure 1 shows the retention of thrombin inhibitory activity of hirudo peptides across various hydrolysis durations.

According to the results, the hirudo extract retained an activity retention rate of 60%–75% after hydrolysis by pepsin. Interestingly, there was a slight decrease in inhibitory activity throughout hydrolysis with the prolongation of hydrolysis time. After 60 min of incubation, the thrombin inhibitory activity showed a slight increase. We speculate that this is due to the generation of new thrombin inhibitory peptides resulting from the hydrolysis of active substances by pepsin. However, after 30 min of incubation with pepsin, the retention of thrombin inhibitory activity of hirudin was only 51.8%. It has also been reported that hirudin is easily degraded in the gastrointestinal tract, leading to a loss of activity (Dong et al., 2016). Enzymatic breakdown poses a hurdle to oral peptide components (Zhu et al., 2021), highlighting the importance of exploring hirudo peptides that exhibit sustained functionality following exposure to gastric proteases. The anticoagulant activity retention rate of HHS after incubation with pepsin was significantly higher than that of hirudin, with a *p*-value less than 0.05. This can be attributed to two reasons: first, the hirudo extract may contain compounds that are resistant to degradation by pepsin; second, the components after degradation still exhibit significant anticoagulant activity. These findings indicate the presence of potent bioactive constituents within the extract that exhibit resistance to enzymatic degradation, thereby maintaining their effectiveness in inhibiting thrombin activity. Further investigation into the specific components responsible for this phenomenon could provide valuable insights into the anticoagulant active ingredients that may be absorbed through oral administration of this extract.

### 3.2 Identification of peptide sequences by nano LC-ESI-MS/MS coupled with de novo sequencing

Given that there was no statistically significant change in the anticoagulant activity of the hirudo extract following incubation with pepsin for 30 and 60 min (*p* > 0.05, with activity being higher at 60 min compared to 30 min) based on the results presented in Section 3.1, and considering the average gastric emptying time of approximately 30 min in healthy individuals (Wu et al., 2022), the peptide components from the HHS after 30 min of incubation were selected for the identification of peptide sequences.

In the present investigation, the PEAKS studio software was employed for its consistent provision of more accurate peptide sequences and higher confidence levels, attributes due to the global optimization algorithms and sophisticated scoring schema utilized by PEAKS(Ma et al., 2003; Zhang et al., 2012). The average local confidence level (ALC %) was defined as the mean of the confidence levels assigned to the presence of a specific amino acid at a given position within the *de novo* sequence (Hou et al., 2019). The present study establishes a threshold of 80% for the Ascore Local Confidence (ALC) value. Peptides meeting this criterion were selected for subsequent analysis (see Section 3.3).

A total of 90 peptides (shown in Supplementary Table S1) were identified at this stage, consisting of amino acid residues ranging from 4 to 21 and molecular weights between 450 and 2,200 Da. This comprehensive approach to peptide identification eliminates the need for traditional fractionation and purification steps.

The proportion of each amino acid residue was obtained by dividing its number of occurrences by the total number. Leu, Gly, Pro, Val, and Asp were the most commonly observed amino acids identified in the HHS (Table 1). Notably, the presence of hydrophobic amino acids (Val, Leu, and Pro) can enhance the thrombin inhibitory activity of polypeptides (Indumathi and Mehta, 2016; Zhang, 2016).

**TABLE 1:** Amino acid composition of HHS.

Amino acid	Number of residues	Percentage (%)
Leu	168	18.4
Gly	99	10.8
Pro	72	7.9
Val	66	7.2
Asp	66	7.2
Glu	64	7.0
Ser	54	5.9
Ala	49	5.4
Phe	45	4.9
Thr	42	4.6
Lys	34	3.7
Tyr	31	3.4
Trp	29	3.2
Asn	28	3.1
Gln	27	3.0
Arg	20	2.2
His	13	1.4
Met	5	0.5
Cys	3	0.3

The peptide hirudin possesses a negatively charged C-terminus and a hydrophobic N-terminus. These distinctive structures may favor binding to thrombin, which typically carries a positive charge, thus exerting a positive effect on its anticoagulant activity (Zhang, 2016). The proportion of peptides from the HHS with the above structural features was 58.9%, indicating that HHS may have anticoagulant effects. Overall, Studies on hirudo pepsin hydrolysates contribute to the expansion of the database of active thrombin inhibitory peptides.

### 3.3 Molecular docking

In the scoring of HPEPDOCK, lower scores indicate higher affinity between the peptide and protein (Zhou et al., 2018). We observe that the scores of these peptides with proteins range between −100 and −200, with twenty-eight percent of the peptides scoring below −180. This indicates that they may exhibit a very strong affinity for the proteins. The hirudin polypeptide fragment (GFFEEIPEEYLQ) was selected as a positive control (Payne et al., 1991). The average docking score for the positive control was −187.184, which demonstrating the validity of our docking methodology. According to the results of HPEPDOCK and literature reports, TYS18 in the hirudin fragments was defined as the active site (Feng et al., 2018). Therefore, we further employed ADCP for local semi-flexible molecular docking at this active site. In the scoring of ADCP, similarly, lower scores indicate higher affinity between the peptide and protein (Zhang and Sanner, 2019). Finally, we normalized the scores obtained from HPEPDOCK and ADCP. The normalized scores range from 0 to 1, where higher scores indicate higher affinity between the peptide and protein. Based on the normalized results, the top ten docking scores (shown in Table 2) were screened out for the next ADMET study to predict absorption and toxicity.

**TABLE 2:** Results of thrombin-peptide docking and ADMET.

Sequence	Length	ALC	HPEPDOCK score	ADCP Score	Normalised score	GI
NDLWDQGLVSQDL	13	81	−205.402	−20.94	1.56	0.93
TPGTVSDLYHFPWQ	14	88	−195.662	−19.37	1.35	0.91
KGGGVQRLSETWPWQ	15	81	−200.314	−22.85	1.63	0.88
VLDESGSLWGPHF	13	97	−220.635	−18.9	1.59	0.88
PLENYLDTEGDAALLDGH	18	93	−189.948	−21.4	1.42	0.87
YSVFDLGGNGKHPP	14	82	−203.627	−18.95	1.41	0.83
LAYPSLSASQTAFFDNL	17	96	−201.365	−20.49	1.49	0.79
VNLSDTNQLFGNLDAYPGSF	20	83	−188.138	−21.61	1.41	0.79
LGDEPLENYLDTEY	14	94	−189.298	−20.64	1.36	0.69
EAAAAALLEPGFPWQ	15	80	−201.706	−17.65	1.30	0.85

Drug discovery is a costly and time-consuming process, and an effective approach to achieving this goal is through Computer-Aided Drug Design (CADD) in the preclinical stages of drug discovery (Shaker et al., 2021; Bassani and Moro, 2023). Among the various methods, virtual screening is a promising CADD technology. Within virtual screening, molecular docking methods have been widely utilized to evaluate the binding potential of each structure with a specific target, thereby improving the overall efficiency of basic drug discovery research (Saikia and Bordoloi, 2019; Sivakumar et al., 2020). Nonetheless, reliance solely on a single molecular docking method can introduce false positives, as the sampling algorithm might fail to produce accurate binding conformations, or the scoring function may not accurately discern the correct binding conformations after scoring and ranking (Vidal-Limon et al., 2022). Using different molecular docking software can enhance the accuracy of molecular docking results. In the peptide-protein docking procedure, it has been shown that HPEPDOCK demonstrates the best performance in global docking, while ADCP yields the most accurate predictions in local docking (Weng et al., 2020). Thus, we employ an innovative approach by utilizing both HPEPDOCK and AutoDock CrankPep docking methods to assess the binding affinity between the hirudo peptide and thrombin, thereby enhancing result accuracy.

### 3.4 ADMET

In this study, we aim to screen for anticoagulant peptides from HHS that have potential for gastrointestinal absorption. Therefore, we conducted absorption and toxicity predictions on the top ten ranked peptide segments from the molecular docking results, as shown in Table 2. In the new drug discovery process, absorption parameters mainly include water solubility, gastrointestinal (GI) absorption, skin permeability, and Caco-2 permeability. When the GI value is greater than 30%, it indicates that the drug molecule has good absorption and that most of the compounds have good absorption (Liu et al., 2022). The gastrointestinal absorption of NDLWDQGLVSQDL (P1) is the highest, reaching over 90%, indicating that it is most likely to be absorbed; thus, it was selected for the subsequent molecular dynamics simulations. In the process of drug development, toxicity is a critical criterion. The selected drug candidates require not only high activity but also have low toxicity. Therefore, toxicity plays an important role in selecting the most suitable drug candidates (Liu et al., 2022). The LD50 (median lethal dose) of the candidate drugs was also predicted using the admetSAR 2.0 online server, and the predicted results show that P1 shows low toxicity.

### 3.5 The thrombin inhibitory activity of P1

**FIGURE 2:**
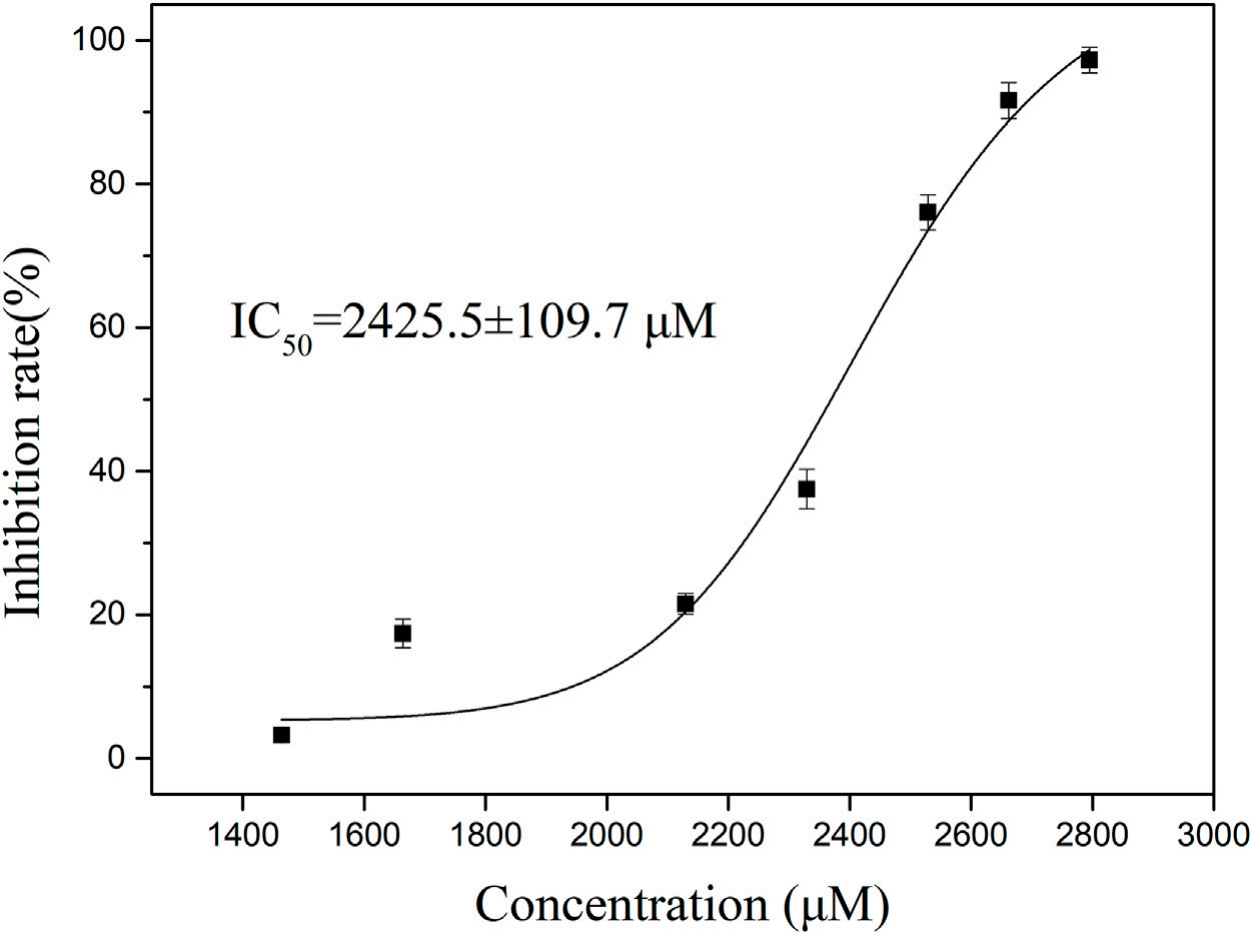
The thrombin inhibitory activity of P1. To determine the inhibition rate of P1 against thrombin, the concentrations of P1 were set ranging from 1,500 to 2,800 μM. The data presented are the means ± SD (n = 3). The black solid curves passing through the data points represent the best fit as determined by logistic analysis, yielding an IC_50_ value of 2,425.5 ± 109.7 μM for P1.

It was synthesized for subsequent analysis to study the inhibitory effect of P1 on thrombin activity, we used different concentrations of P1 to verify the inhibitory effect of P1 on thrombin (Figure 2). The results showed that the inhibitory effect of P1 on prothrombin activity was dose-dependent. The data were analyzed using the logistic analysis program in OriginPro 9.0, resulting in an IC_50_ = 2,425.5 ± 109.7 μM.

Hirudin is a highly specific and potent inhibitor of thrombin, binding to the enzyme with high affinity (Dong et al., 2016). However, its use in therapeutic settings is limited by instability in the gastrointestinal tract, which precludes oral administration. Although P1 did not exhibit the potent thrombin inhibitory activity as expected, it is a product of pepsin hydrolysis and holds greater potential for oral absorption. The findings suggest that the selectivity and inhibitory activity of natural peptide P1 still need improvement. Further structural modifications are necessary to improve P1’s pharmacological properties and validate in subsequent studies.

### 3.6 Determination of PT, APTT and TT

**FIGURE 3:**
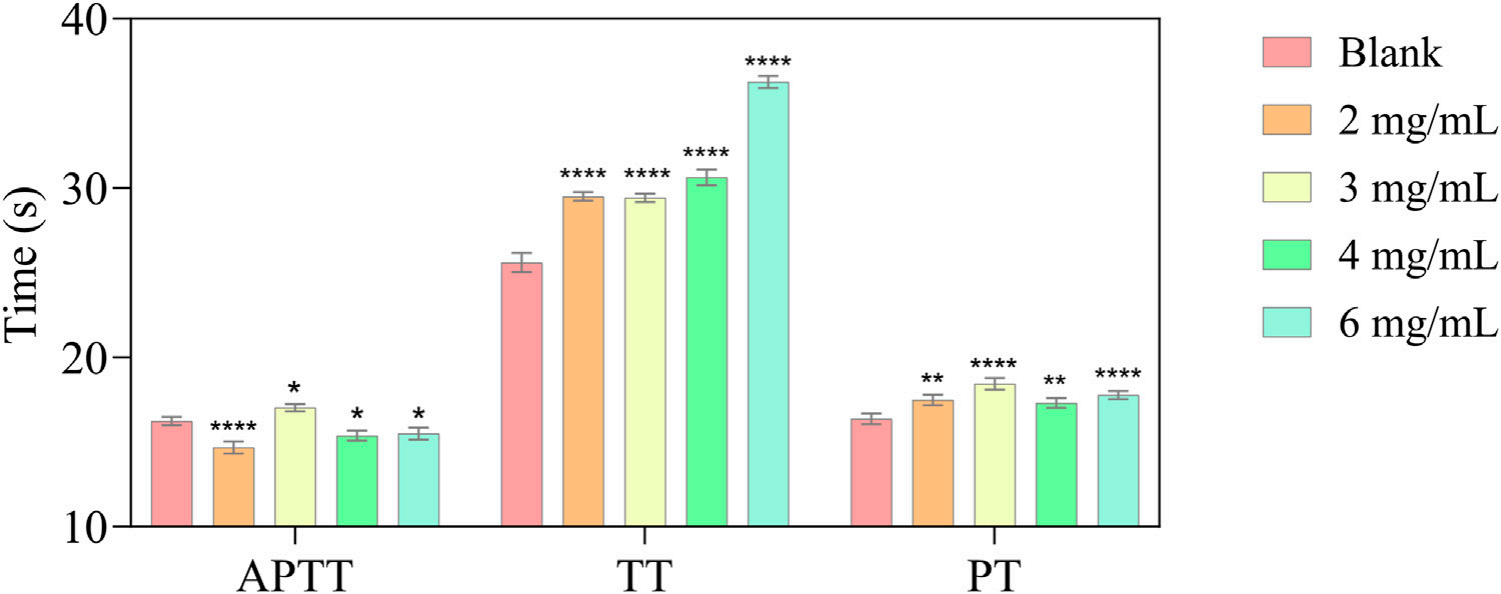
*In vitro* anticoagulant activity of different P1 concentrations (APTT; TT; PT). Values are represented as means ± SD (n = 3). Compared with the blank group, *p < 0.05; **p < 0.01; **** p < 0.0001.

The blood coagulation process consists of the endogenous pathway, exogenous pathway and common pathway. When the coagulation process reaches the point where factor X is activated through the exogenous and endogenous pathways, the two pathways are combined into a common pathway (Gaertner and Massberg, 2024). In the clinical area, activated partial thromboplastin time (APTT), prothrombin time (PT), and thrombin time (TT) are used to evaluate the functions of the aforementioned three pathway (Favaloro et al., 2011). The anticoagulant activity of P1 was evaluated using PT, TT, and APTT. As shown in Figure 3, compared to the control buffer, P1 markedly prolonged TT to 36 s (*p* < 0.0001) in a dose-dependent manner, while it can also significantly prolong PT, but there is no indication of a dose-dependent relationship.

### 3.7 The antiplatelet aggregation activity of P1

**FIGURE 4:**
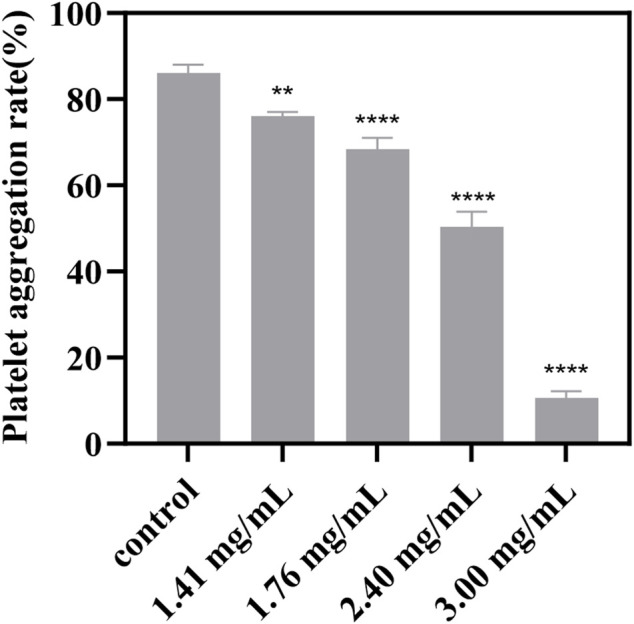
Effect of different concentrations of P1 on ADP-induced platelet aggregation. PRP that was not treated with P1 served as the control. Effect of P1 on ADP-induced platelet aggregation was tested at four different concentrations (*x*-axis). Data are expressed as means ± SD (n = 3). Compared with the control group, *p < 0.05; **p < 0.01; ****p < 0.0001.

In this study, we conducted an ADP-induced platelet aggregation assay to investigate the effect of P1 on platelet aggregation. Figure 4 shows that P1 effectively reduces platelet aggregation induced by ADP in a dose-dependent manner.

Platelet aggregation is a critical step in the pathogenesis of thrombotic diseases, such as myocardial infarction and stroke (Nieswandt et al., 2011). P1 tested in this study has shown promising antiplatelet effects, suggesting potential clinical applications in the prevention and treatment of thrombotic diseases. However, it is important to note that the results of this study are based on *in vitro* experiments, and further studies are needed to investigate the drug’s efficacy and safety *in vivo*.

### 3.8 Interaction assay between thrombin and peptide

**FIGURE 5:**
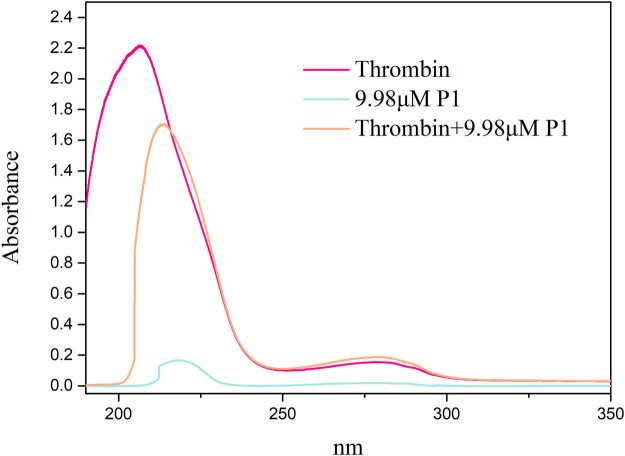
UV-Vis spectra of thrombin plus P1 and thrombin. The *x*-axis displays the wavelength (nm), while the *y*-axis indicates the absorbance. Spectra were obtained for thrombin alone and for complex of thrombin and P1. Key observations include shifts in absorbance peaks, which suggest conformational changes in thrombin upon binding with P1.

UV-Vis absorption measurement is a simple and effective method that can be used to identify structural changes in proteins and investigate the formation of ligand-protein complexes (Cheng et al., 2013). In this study, the UV-Vis spectra of P1 and thrombin both in the presence and absence of P1 were obtained between 190 and 350 nm (Figure 5). Thrombin exhibited two absorption peaks at 206 and 280 nm. When the P1 solution was added to the thrombin solution, the peak at 206 nm was slightly red-shifted (7 nm) and decreased in intensity, likely due to the interaction of P1 with thrombin, which resulted in the disorganization of the surroundings of amide bonds and the contraction of the C=O bonds in thrombin (Chen et al., 2019). These results suggest that there is an interaction between P1 and thrombin and that a complex is formed.

### 3.9 Molecular dynamics simulation analysis of thrombin-peptide complex

To further investigate the potential interaction patterns between P1 and thrombin at an atomic level, we carried out molecular dynamics (MD) simulations.

**FIGURE 6:**
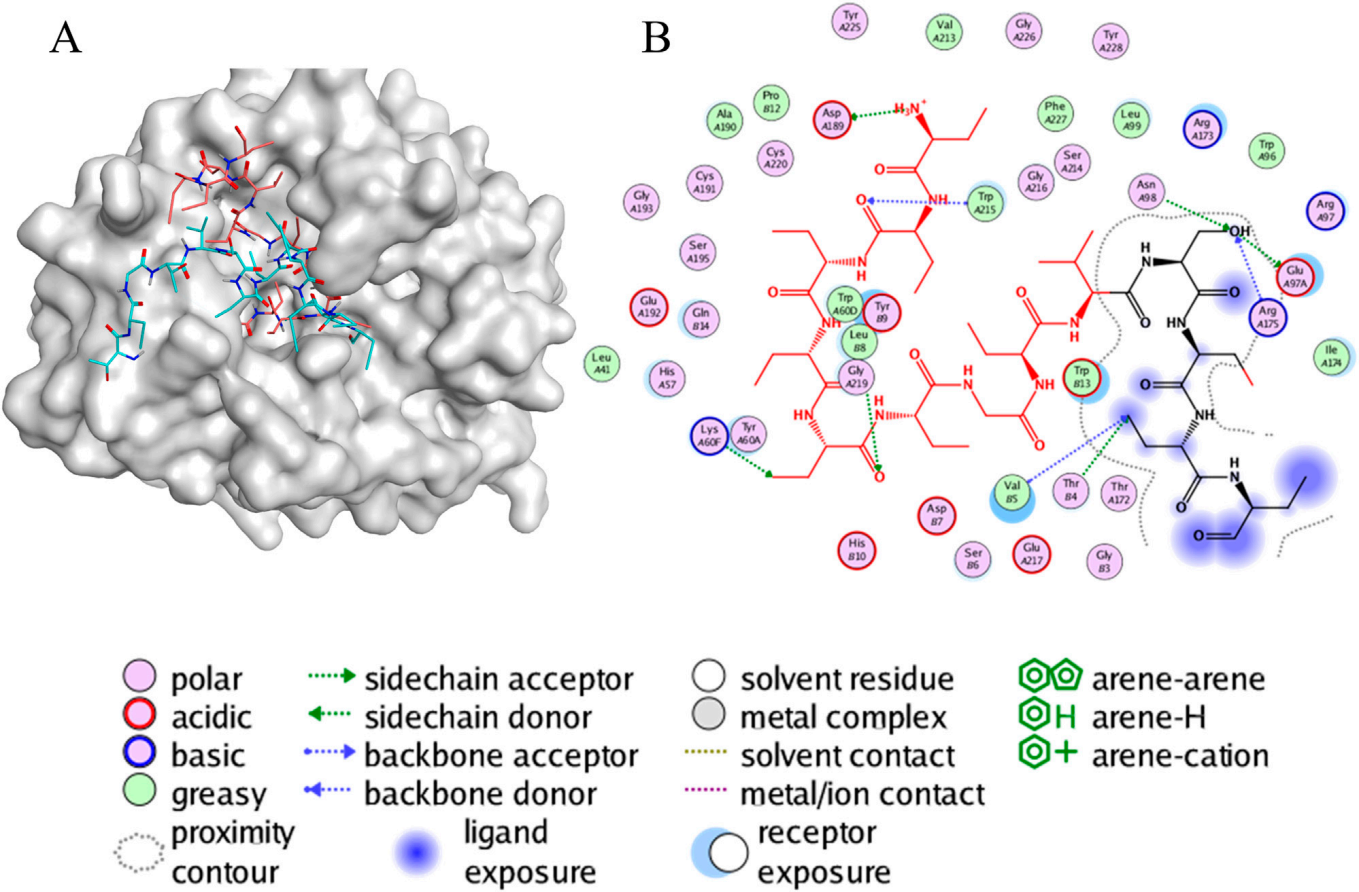
The interaction of peptides and protein. Visualization and graphics were generated with PyMOL software (https://pymol.org/2/support.html) **(A)** A 3D view of molecular docking. This representation depicts the conformation of the peptide-protein complex obtained from ADCP docking, highlighting the spatial arrangement of P1 within the thrombin binding site. **(B)** A 2D view of the interaction between P1 and protein. This diagram outlines the key contacts and interactions, such as hydrogen bonds, which play a crucial role in stabilizing the peptide-protein complex.

First, the conformation of the peptide-protein complex obtained from the ADCP docking was used as the initial conformation for the molecular dynamics simulations. The 3D interaction map of the initial conformation is depicted in Figure 6A, while Figure 6B represents the two-dimensional schematic interactions between P1 and the protein. The interaction between P1 and the protein is predominantly mediated by hydrogen bonding, wherein Asp-A189, serving as an acidic hydrogen bond acceptor, interacts with the amino group in P1, while Trp-A215, Gly-A219, Lys-A60F, Asn-A98, Glu-A97A, Arg-A175, Thr-B4, and Val-B5 serve as hydrogen bond donors.

**FIGURE 7:**
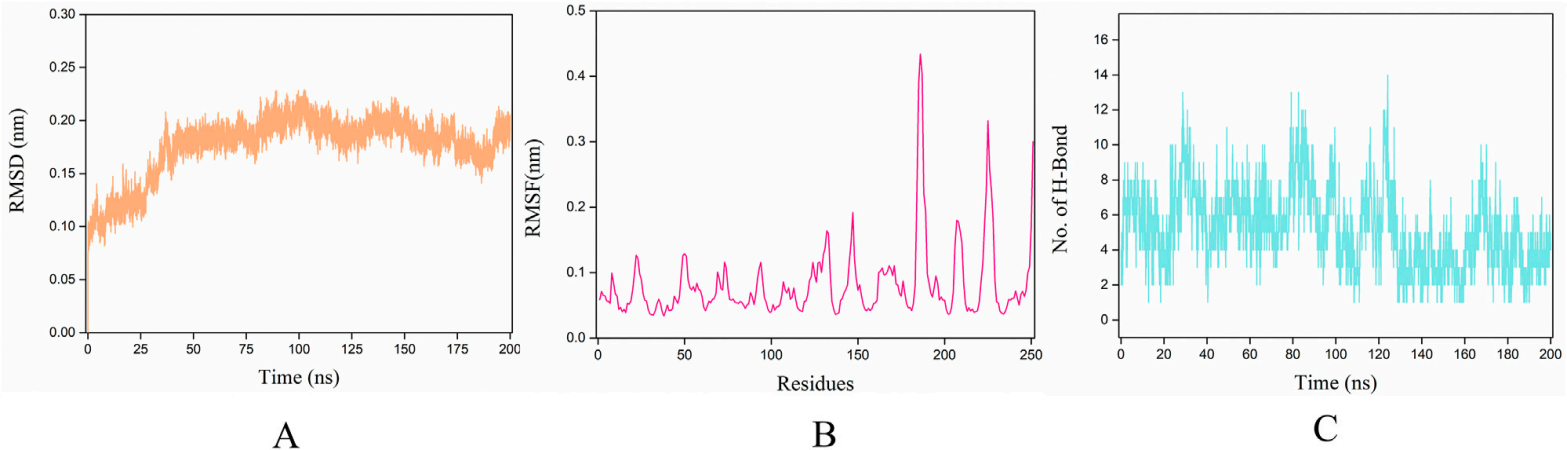
Molecular dynamics simulation results. The x-axis for all three figures represents the simulation time of 200 ns, while the y-axes represent RMSD, RMSF, and H-bond, respectively. **(A)** RMSD changes during the simulation. The Root Mean Square Deviation (RMSD) value over time is often used to check whether a simulation system has reached stability, where lower RMSD values indicate greater stability of the protein-ligand complex. This figure depicts the RMSD of the Cα atoms of thrombin bound to ligands over time during the 200 ns simulations. **(B)** RMSF changes during the simulation. Aiming also at the conformational changes of the protein observed during the MD simulations, the fluctuations of the mean structure of the protein, expressed in terms of fluctuations of the Root Mean Square Fluctuations (RMSF). This figure illustrates the fluctuation curves of the Cα atoms across different amino acid residues. **(C)** H-bond evolution pattern of the thrombin-peptide docked complex, showing that the time-dependent intermolecular hydrogen bonding pattern throughout 200 ns trajectory.

The stability of receptor-ligand complexes and the dynamics of ligand binding were assessed through molecular dynamics simulations that mimicked physiological conditions including solvent presence, specific temperature, and pressure. In our investigation of the conformational stability of thrombin with its ligands (P1), we conducted 200 ns molecular dynamics simulations. Initially, during the 200 ns simulations, we determined the Root Mean Square Deviation (RMSD) of the Cα atoms of thrombin bound to P1 over time. The RMSD trajectories for all thrombin-P1 complexes converged and remained stable, with values below 0.25 nm (Figure 7A). The average RMSD value for the thrombin-P1 complex was 0.175 nm. The RMSD plots depict potential positional fluctuations of the complex, where lower RMSD values indicate greater stability. The results indicate that P1 can maintain stability within the active site.

To investigate the binding of P1 to thrombin, Root Mean Square Fluctuations (RMSF) of the Cα atoms were calculated for each individual amino acid residue. Figure 7B illustrates the fluctuation curves of the Cα atoms for different amino acid residues, with the most residues displaying fluctuations below 0.3 nm. We analyzed residues with fluctuations greater than 0.3 nm, revealing significant fluctuations in ASP-186A, GLU-186B, PRO-186, ASP-221, and GLY-246.

H-bonds are vital intermolecular forces that contribute to complex stability between a protein and its binding partner (van der Lubbe and Fonseca Guerra, 2019). To assess the stability of hydrogen bonds formed between peptide and thrombin, the time-dependent intermolecular hydrogen bonding pattern throughout the 200 ns trajectory was extracted. This analysis showed that P1 formed an average of ∼5.2 H-bonds (with the maximum of 14) with thrombin during the simulation time (Figure 7C). It can be concluded that the peptide and thrombin were hydrogen bonded to each other for a significant amount of time.

The simulation results provided with a detailed view of the potential binding mode between these two molecules. First, we observed that the binding of the peptide to thrombin was stable, with no significant dissociation. This indicates a strong interaction between the peptide and thrombin, favoring its anticoagulant effects *in vivo*. Additionally, the peptide exhibited minimal conformational changes during the binding process, suggesting that it can maintain a stable structure beneficial for its sustained anticoagulant activity. Second, through the analysis of the interaction forces during the simulation, we found that hydrogen bonds are primarily maintain the binding of the peptide to thrombin. This suggests that modifying the peptide’s amino acid sequence to enhance these interaction forces could potentially improve its inhibitory activity against thrombin (Cabrele et al., 2014). Furthermore, we observed changes in key amino acids associated with thrombin activity during the simulation. These alterations may affect thrombin’s activity, providing further evidence of the peptide’s inhibitory effect. In conclusion, molecular dynamics simulations have provided a deeper understanding of the interaction between thrombin and the peptide, revealing important clues about its anticoagulant mechanism. These findings lay the foundation for future research and drug development, with the potential to offer safer and more effective anticoagulant therapies in clinical settings.

## 4 Conclusion

The oral route is the oldest and most widely employed method for drug delivery (Sastry et al., 2000). In traditional Chinese medicine, leeches are typically administered orally after drying, processing, and boiling (Dong et al., 2016). Oral administration involves passage through the gastrointestinal tract, where digestive enzymes degrade substances. In theory, proteins or peptides from leeches may undergo structural changes during these processes, losing their activity, much like hirudin. Thus, the active peptides released from processed leech digestive enzyme hydrolysates are more likely to be the active ingredients absorbed by the human body when administered orally. In our preliminary research, we found that hirudo extract still exhibited significant anticoagulant activity even after incubation with gastric protease. As a result, we proceeded with its component identification. A total of 90 peptides were first identified from the gastric protease hydrolysate of hirudo extract, among which one novel bioactive peptide, NDLWDQGLVSQDL, was screened and evaluated using a more efficient and comprehensive strategy, which complements the database of potentially active peptides in CVD. The novel peptide NDLWDQGLVSQDL identified in this study has shown significant thrombin-inhibitory activity, making it a promising candidate for developing anticoagulant therapies, particularly for conditions like deep vein thrombosis, pulmonary embolism, and other cardiovascular diseases. Its simple structure allows for easier chemical synthesis and modification, enabling potential improvements in potency, stability, and bioavailability. Beyond its role as an anticoagulant, this peptide could have wider applications in biotechnology and pharmaceuticals, such as in diagnostic tools or medical device coatings to prevent blood clots. However, further *in vivo* studies are needed to confirm its efficacy and safety for clinical use.

This research introduces a more comprehensive and scientific system for screening thrombin inhibitors in HHS through the innovative integration of HPEPDOCK and ADCP docking methods, which incorporates the assessment of absorption probabilities. A comprehensive evaluation system was employed, which included determining its IC_50_ value and validating its anticoagulant effects (TT, APTT, PT) and anti-platelet aggregation activity. Furthermore, the strong binding affinity of this peptide to thrombin was demonstrated through UV-Vis spectroscopy and molecular dynamics simulations. However, there are certain limitations to this study that should be considered. First, while the peptide P1 exhibited thrombin inhibitory activity, its IC_50_ value was relatively high compared to other inhibitors reported in the literature (Huang et al., 2020). This suggests that while P1 holds potential, further optimization may be necessary to enhance its potency. Additionally, the study primarily focused on *in vitro* conditions, and future research will need to assess the *in vivo* efficacy and stability of the peptide in physiological environments. Finally, although simulation techniques provided valuable mechanistic insights, experimental validation of these simulations in more diverse biological systems is required to confirm the peptide’s therapeutic potential. On the whole, this study provides new research ideas and a material basis for the development of new drugs for CVD.

## Data Availability

The original contributions presented in the study are publicly available. This data can be found here: https://figshare.com/articles/dataset/peptide_sequences/27683049/1?file=50408013.
