# Supplementary material for: Identification, screening, and comprehensive evaluation of novel thrombin inhibitory peptides from the hirudo produced using pepsin
Source: Front Pharmacol. 2024 Nov 21;15:1460053. doi: 10.3389/fphar.2024.1460053 (PMC11617586; doi:10.3389/fphar.2024.1460053)
Supplement: Supplementary file 1 [file Table1.DOCX]

***Supplementary Table S1 Peptides from the HHS identified by nano LC-MS/MS and Molecular Docking Score.***

| Peptide sequence | Retention  time  (min) | Mass | ALC | HPEPDOCK  score | ADCP  Score | Normalised score |
| --- | --- | --- | --- | --- | --- | --- |
| YYTVF | 50.49 | 691.3217 | 99 | -180.597 | -15.13 | 0.88857 |
| WVPSVYC(+57.02)SSL | 53.53 | 1196.554 | 99 | -205.969 | -15.82 | 1.220397 |
| LGDEPLENYYSTEY | 56.52 | 1691.72 | 99 | -180.183 | -19.14 | 1.155599 |
| LGGGGKLPDLDLGGGLK | 44.19 | 1565.878 | 99 | -174.579 | -22.06 | 1.290465 |
| LLAPP | 28.29 | 509.3213 | 99 | -132.86 | -10.24 | 0.020899 |
| WNVPVSVEGY | 53.85 | 1148.55 | 98 | -178.111 | -17.74 | 1.037467 |
| LDRVFDL | 45.5 | 876.4705 | 98 | -149.533 | -16.56 | 0.63642 |
| LRWLL | 54.68 | 699.4432 | 98 | -179.472 | -15.67 | 0.912515 |
| KVFYENL | 40.78 | 911.4752 | 98 | -169.933 | -17.35 | 0.919157 |
| LGGGGKLPDLDLNGLLK | 54 | 1678.962 | 98 | -148.435 | -21.99 | 0.991969 |
| WLLGDV | 56.92 | 701.3748 | 97 | -164.276 | -15.48 | 0.728901 |
| VLDESGSLWGPHF | 54.35 | 1442.683 | 97 | -220.635 | -18.9 | 1.593856 |
| AAPGEFPW | 55.07 | 873.402 | 97 | -183.693 | -13.9 | 0.840024 |
| K(+42.01)GLGGGGKLPDLDL | 53.56 | 1380.761 | 97 | -147.056 | -20.12 | 0.849781 |
| WVPSVY | 47.4 | 749.3748 | 96 | -203.567 | -14.18 | 1.082297 |
| LAYPSLSASQTAFFDNL | 57.71 | 1843.899 | 96 | -201.365 | -20.49 | 1.485062 |
| VLDESGSLWGPFH | 54.35 | 1442.683 | 96 | -186.477 | -18.97 | 1.2148 |
| EDGLPLENYL | 55.17 | 1161.555 | 96 | -149.31 | -17.39 | 0.690148 |
| WLVPSVY | 56.39 | 862.4589 | 96 | -208.431 | -16.46 | 1.29142 |
| PDLGGGLGGLGGGGKLDPLDL | 56.26 | 1876.989 | 96 | -151.7 | -22.2 | 1.042882 |
| LGDEPLENYLDTEY | 56.52 | 1669.736 | 94 | -189.298 | -20.64 | 1.359641 |
| FTVLF | 57.77 | 625.3475 | 94 | -159.097 | -13.75 | 0.553502 |
| PLENYLDTEGDAALLDGH | 59.17 | 1941.896 | 93 | -189.948 | -21.4 | 1.418435 |
| TLLEVE | 38.04 | 702.3799 | 93 | -137.755 | -14.33 | 0.352999 |
| LTVLGNE | 29.94 | 744.4017 | 93 | -154.96 | -15.03 | 0.593739 |
| K(+42.01)FPSLV | 54.22 | 731.4218 | 92 | -157.657 | -13.61 | 0.527837 |
| FLGGHL | 33.18 | 642.3489 | 92 | -170.468 | -14.41 | 0.725981 |
| SVWLNSL | 53.06 | 817.4334 | 92 | -177.977 | -15.58 | 0.88962 |
| TVLFFDTEVMV | 56.56 | 1299.642 | 91 | -177.487 | -18.34 | 1.071106 |
| SYVGDEAQSKARVL | 31.44 | 1521.779 | 91 | -166.614 | -20.74 | 1.111539 |
| EGFPWQ | 52.63 | 762.3337 | 91 | -189.88 | -14.43 | 0.945448 |
| EEPWQ | 59.46 | 687.2864 | 90 | -158.696 | -12.94 | 0.494118 |
| WDQGLVSQDN | 50.78 | 1160.51 | 90 | -174.302 | -18.35 | 1.035997 |
| FSVY | 37.12 | 514.2427 | 90 | -152.491 |  | 0.241472 |
| TM(+15.99)ELDRLEDEL | 48.19 | 1378.629 | 89 | -171.932 | -19.97 | 1.119124 |
| REPSVY | 47.4 | 749.3708 | 89 | -168.611 | -14.01 | 0.678016 |
| FSEEGALKAGQGLLGL | 52.96 | 1588.846 | 88 | -158.311 | -22.28 | 1.122583 |
| LSLFDTGSSNL | 48.32 | 1152.566 | 88 | -175.142 | -17.18 | 0.966167 |
| LTGFGLGTPAQDF | 55.17 | 1322.651 | 88 | -162.694 | -18.17 | 0.893375 |
| TPGTVSDLYHFPWQ | 56.62 | 1646.773 | 88 | -195.662 | -19.37 | 1.345103 |
| LLGDVF | 53.13 | 662.3639 | 88 | -149.999 | -14.74 | 0.51835 |
| KLFDEF | 44.6 | 797.3959 | 88 | -157.523 | -16.68 | 0.734326 |
| FLLP | 51.98 | 488.2998 | 88 | -146.673 |  | 0.176101 |
| V(+42.01)SREAEAALEQEEAKVKYLAH | 56.08 | 2412.228 | 88 | -156.01 | -25 | 1.281011 |
| ETDLYPQPLGLGTPAQDF | 59.49 | 1960.942 | 87 | -171.44 | -18.17 | 0.991645 |
| KLKLLAEPPKR | 16.92 | 1291.834 | 87 | -170.83 | -19.73 | 1.090482 |
| FAALFDEEPPAAP | 55.7 | 1373.65 | 87 | -161.613 | -13.97 | 0.596676 |
| TLSLLT | 42.55 | 646.3901 | 87 | -148.845 | -14.01 | 0.455926 |
| EVM(+15.99)QVP | 44.14 | 717.3367 | 87 | -146.511 | -12.51 | 0.328075 |
| LGQDWVSQDL | 50.82 | 1159.551 | 86 | -172.215 | -18.24 | 1.005095 |
| LSETKDAKFPVP | 33.41 | 1330.713 | 86 | -192.617 | -18.04 | 1.220781 |
| KYFLL | 50.55 | 682.4053 | 86 | -169.637 | -15.82 | 0.812172 |
| EDGLALPLENYLDTEY | 59.17 | 1853.857 | 85 | -166.674 | -21.83 | 1.186062 |
| LGLEGL | 47.89 | 600.3483 | 85 | -131.525 | -13.63 | 0.235574 |
| TVLFDTGSSNL | 48.32 | 1152.566 | 85 | -155.646 | -17.54 | 0.771501 |
| VTQVGGLSDTNQLFGL | 57.77 | 1647.847 | 85 | -178.052 | -19.97 | 1.187888 |
| LRLL | 32.72 | 513.3638 | 85 | -138.655 |  | 0.086011 |
| TVPSASGATPVQPGGF | 53.92 | 1471.731 | 85 | -177.857 | -14.53 | 0.817134 |
| TVGGGGLWDDHLC(+57.02)L | 56.07 | 1498.688 | 85 | -171.439 | -20.1 | 1.122392 |
| TVGLNW | 45.36 | 688.3544 | 85 | -166.039 | -14.91 | 0.710092 |
| DDQVVSEKDYSHLRL | 42.68 | 1802.88 | 84 | -170.316 | -22.72 | 1.287281 |
| TVELVE | 37.6 | 688.3643 | 84 | -139.268 | -13.71 | 0.327994 |
| TLLGNE | 24.18 | 645.3333 | 84 | -134.629 | -13.78 | 0.280613 |
| ELHAAAP | 44.41 | 707.3602 | 84 | -145.87 | -12.52 | 0.32155 |
| TLYFP | 38.62 | 639.3268 | 84 | -173.738 | -13.36 | 0.691584 |
| VNLSDTNQLFGNLDAYPGSF | 57.71 | 2171.017 | 83 | -188.138 | -21.61 | 1.412325 |
| LRDLAGRDTVEYL | 53.72 | 1519.799 | 83 | -183.666 | -20.58 | 1.292295 |
| LGRLPQVL | 41.4 | 894.5651 | 83 | -167.713 | -15.54 | 0.771584 |
| PPVSEDWVYPEPLDW | 57.34 | 1827.835 | 82 | -190.992 | -18.09 | 1.20591 |
| AFGAC(+57.02)KNDPFVR | 33.5 | 1380.661 | 82 | -189.145 | -17.84 | 1.16822 |
| YSVFDLGGNGKHPP | 56.03 | 1486.721 | 82 | -203.627 | -18.95 | 1.406142 |
| TVGEAAFGLKENGAGP | 52.63 | 1516.752 | 82 | -159.041 | -19.15 | 0.918726 |
| RPKHPR | 8.66 | 789.4722 | 82 | -178.739 | -14.24 | 0.807396 |
| WKVKRADL | 17.66 | 1014.597 | 81 | -160.509 | -20.39 | 1.019231 |
| WNVPSPPKLN | 54.02 | 1150.614 | 81 | -203.288 | -15.95 | 1.199081 |
| KGGGVQRLSETWPWQ | 56.62 | 1727.874 | 81 | -200.314 | -22.85 | 1.633145 |
| LLLF | 56.95 | 504.3311 | 81 | -138.297 |  | 0.081989 |
| PSLSASHGGELTDGGL | 53.95 | 1496.711 | 81 | -179.103 | -19.21 | 1.148207 |
| NDLWDQGLVSQDL | 57.57 | 1501.705 | 81 | -205.402 | -20.94 | 1.56091 |
| KDWM(+15.99)VP | 54.48 | 790.3683 | 81 | -172.363 | -15.51 | 0.821799 |
| TASSSVP | 36.79 | 647.3126 | 81 | -140.823 | -11.38 | 0.187607 |
| T(+42.01)LDKFLP | 42.79 | 874.48 | 81 | -154.961 | -15.9 | 0.652694 |
| ASNGGLLSPGEFPWQ | 54.88 | 1558.742 | 80 | -196.847 | -17.36 | 1.222239 |
| LRVL | 29.73 | 499.3482 | 80 | -165.953 |  | 0.39273 |
| PWAGL | 48.06 | 542.2853 | 80 | -174.254 | -12.8 | 0.659442 |
| LRDLDFKSPGPYL | 53.72 | 1519.804 | 80 | -187.601 | -19.76 | 1.280953 |
| LVLRNVL | 39.83 | 825.5436 | 80 | -165.379 | -16.44 | 0.806335 |
| K(+42.01)EELWKVVHELED | 29.4 | 1694.852 | 80 | -149.714 | -23.12 | 1.082898 |
| EAAAAALLEPGFPWQ | 58 | 1569.783 | 80 | -201.706 | -17.65 | 1.296482 |
| MALTT | 49.59 | 535.2676 | 80 | -132.68 | -11.27 | 0.08866 |

(+57.02) represents a Carbamidomethylation posttranslational modification

(+15.99) represents a Oxidation posttranslational modification.

(+42.01) represents a Acetyl posttranslational modification
